# Emergency care of sepsis in sub-Saharan Africa: Mortality and non-physician clinician management of sepsis in rural Uganda from 2010 to 2019

**DOI:** 10.1371/journal.pone.0264517

**Published:** 2022-05-11

**Authors:** Brian Rice, Sal Calo, John Bosco Kamugisha, Nicholas Kamara, Stacey Chamberlain, on behalf of Global Emergency Care Investigator Study Group

**Affiliations:** 1 Department of Emergency Medicine, Stanford University, Palo Alto, California, United States of America; 2 Global Emergency Care, United States of America; 3 Department of Emergency Medicine and Center for Global Health, University of Illinois at Chicago, Chicago, Illinois, United States of America; 4 Department of Pulmonary and Critical Care Medicine, Respiratory Institute—Cleveland Clinic, Cleveland, Ohio, United States of America; 5 Karoli Lwanga Hospital, Nyakibale, Uganda; Indiana University School of Medicine, UNITED STATES

## Abstract

**Introduction:**

Little data exists from sub-Saharan Africa describing incidence and outcomes of sepsis in emergency units and uncertainty exists surrounding optimal management of sepsis in low-income settings. There exists limited data regarding quality care metrics for non-physician clinicians trained in emergency care. The objective of this study was to describe changes in septic patients over time and evaluate associations between sepsis care and mortality.

**Methods:**

Secondary analysis of a prospective cohort of all consecutive patients seen from 2010–2019 in a rural Ugandan emergency unit staffed by non-physician clinicians was performed using an electronic database based on paper charts. Sepsis was defined as suspected infection with a quick Sequential Organ Failure Assessment score (qSOFA)≥1. Multi-variable logistic regression was used to analyze three-day mortality.

**Results:**

Overall, 48,653 patient visits from 2010–2019 yielded 17,490 encounters for patients age≥18 who had suspected infection, including 10,437 with sepsis. The annual proportion of patients with sepsis decreased from 45.0%% to 21.3% and the proportion with malarial sepsis decreased from 17.7% to 2.1% during the study period. Rates of septic patients receiving quality care (“both fluids and anti-infectives”) increased over time (21.2% in 2012 to 32.0% in 2019, p<0.001), but mortality did not significantly improve (4.5% in 2012 to 6.4% in 2019, p = 0.50). The increasing quality of non-physician clinician care was not associated with reduced mortality, and treatment with “both fluids and antibiotics” was associated with increased mortality (RR = 1.55, 95%CI 1.10–2.00).

**Conclusion:**

The largest study of sepsis management and outcomes ever published in both Uganda and sub-Saharan Africa showed sepsis and malarial sepsis decreasing from 2010 to 2019. The increasing quality of non-physician clinician care did not significantly reduce mortality and treatment with “both fluids and antibiotics” increased mortality. With causal associations between antibiotics and mortality deemed implausible, associations between sepsis mortality and interventions likely represent confounding by indication. Defining optimal sepsis care regionally will likely require randomized controlled trials.

## Background

Sepsis is defined as life-threatening organ dysfunction caused by a dysregulated host response to infection, while septic shock is a subset of sepsis in which there are underlying circulatory, cellular, and metabolic abnormalities that are associated with an even greater risk of mortality [1]. Sepsis is one of the most significant worldwide causes of morbidity and mortality, with 48.9 million annual cases and 11 million annual deaths (19.7% of all global deaths) according to the most recent analysis of the Global Burden of Disease Study from 2017 data [2]. Sepsis disproportionately affects persons in low-and-middle income countries (LMICs) [2, 3]. The World Health Organization has identified sepsis as an international priority, adopting a resolution in 2017 to improve the prevention, diagnosis, and clinical management of sepsis [4]. While in-hospital mortality rates can be greater than 25–30% across resource settings, mortality rates of up to 38% are reported in LMICs, particularly in patients who have confirmed bacteremia [1, 3, 5]. In Uganda, a low-income country in sub-Saharan Africa (SSA), the primary causes of death are all infectious in nature–namely malaria, HIV/AIDs, pneumonia, tuberculosis and diarrhea–and all can be associated with sepsis. Inpatient mortality rates have been reported between 34–43% [6–9].

Identifying patients with sepsis and septic shock presents a significant challenge. Clinical criteria to identify patients with these syndromes have evolved over time. The most recent Sepsis-3 consensus recommendations suggest using bedside clinical criteria, termed the quick Sequential Organ Failure Assessment (qSOFA) score [1]. Adult patients with suspected infection and two or more of the following findings are determined to meet sepsis criteria: altered mentation, respiratory rate ≥ 22, and systolic blood pressure ≤ 100. Using the qSOFA score in a low-resource setting is particularly useful because it does not rely on advanced diagnostic testing. It is a concise, objective scale which provides opportunity for a rapid objective clinical evaluation of the patient by a variety of providers including nurses, mid-level providers, and physicians. While qSOFA was developed in high-income settings, recent studies have validated its usage in LMICs, but the authors have suggested that using a score of one qSOFA criteria (moderate risk of death) is more appropriate than two (high risk of death) for early identification of patients at increased risk of in-hospital mortality in low-resource settings [10, 11].

Management of sepsis globally has changed significantly over the past two decades. In the US and other high-resource settings, “early goal-directed therapy” sparked widespread interest and change in practice patterns for sepsis management, while subsequent trials have called into question the need for higher-resourced aspects of the protocol [12–17]. To add to the confusion, a landmark study in children in SSA showed that fluid boluses, a cornerstone of sepsis management in high resource settings, increase instead of decrease mortality [18]. Some subsequent studies showed similar increased mortality with fluids [19, 20]. However, most experts agree that management should focus early antibiotics and source control plus some combination of fluid resuscitation and early initiation of vasopressor therapy, tailored to the patient presentation.

Emergency medicine is rapidly developing in SSA generally and Uganda specifically [21–23]. With this development, initial sepsis care is shifting to the emergency unit and a better understanding of the burden and outcomes of sepsis from an emergency medicine perspective is required. Sepsis guidelines specific to emergency care in SSA are rare, with the African Federation of Emergency Medicine Handbook serving as the most definitive reference with its recommendations for fluids and early antibiotics following the Surviving Sepsis Campaign [24]. The objective of the following study is twofold: to describe the how the characteristics of patients presenting with sepsis to a rural Ugandan emergency unit data changed from 2010 to 2019 and analyze the impact of sepsis care with fluids and anti-infectives by non-physician clinicians on emergency unit sepsis mortality. This evidence base for emergency unit sepsis care will contribute to the understanding of larger trends in sepsis over time in Uganda, the ability of non-physician clinician training programmes to impact emergency care quality, and the impact of sepsis management on mortality.

## Methods

### Description of study site

All data comes from the emergency unit at Karoli Lwanga Hospital, a rural district hospital located outside the town of Rukungiri (population 32,000) in the Rukungiri District (population 330,000) of southwest Uganda. Rukungiri District is largely agricultural district and lacks a regional referral hospital. With the closest regional referral hospital 120km away in Mbarara, Karoli Lwanga is one of two private non-profit hospitals that serve as district hospitals providing the highest levels of care in the district [25, 26]. The hospital has 200 beds and a six-bed emergency unit that opened in 2008 and treats 300 to 700 patients per month arriving between 8:00 am and midnight every day of the year. Since 2010, the emergency unit has been staffed independently by non-physician clinicians called emergency care practitioners (ECPs) who received training from physicians working with Global Emergency Care, a Uganda- and US-based non-governmental organization. The ECPs are nurses who have completed a two-year advanced training course in emergency care described in detail elsewhere [22]. While training from 2008 to 2010 was directly supervised by visiting US emergency medicine physicians, ECPs have practiced independent care without direct physician supervision since 2010 and with subsequent training of new cadres in the pilot phase of the project. Though there is not any real-time physician supervision of clinical care, although the ECPs consult local physicians for patients who require surgery, do not respond to initial treatments, or in whom there is considerable diagnostic uncertainty. Emergency unit patients were admitted to medical and surgical wards staffed by Ugandan physicians with standard levels of training and no connection to Global Emergency Care.

During the study period, the hospital lacked critical care units, ventilators, capabilities for invasive monitoring, and vasopressor medications (other than epinephrine vials for intended management of anaphylaxis). Diagnostic testing was limited (described below) and radiology (X-ray and ultrasound) was limited with variable availability. Over time, some additional testing including basic metabolic panels became available, and ECPs adopted bedside ultrasonography. Resource utilization by clinicians in this emergency unit is described in detail elsewhere [27].

### Data collection

Global Emergency Care maintained a prospectively collected quality assurance database of all Karoli Lwanga Hospital emergency unit patient visits. Data collected on all patients included: demographics, chief complaint, vital signs (heart rate, respiratory rate, temperature, oxygen saturation and blood pressure), clinician impression of patient initial clinical status, medications administered (intravenous and oral), procedures performed, diagnostic testing (hemoglobin, blood grouping, urinalysis, blood smear for malaria, fingerstick glucose, cerebrospinal fluid analysis, basic metabolic panels and HIV rapid testing), radiology results (X-ray, ultrasound), diagnosis, disposition (admit, discharge, direct to theatre, expired in the emergency unit, referred, left against medical advice, eloped) as well as follow-up vital status (mortality) for all admitted and discharged patients. On Day 3 following initial evaluation in the emergency unit, patients admitted to the hospital were visited in person, while patients discharged from the emergency unit or ward were contacted via phone when available. A rigorous follow-up protocol which included seven consecutive days of calling patients on the phone before considering them lost to follow-up was used for all patients, and this protocol is described in detail elsewhere [22]. When the database was developed, three-day follow-up was chosen to minimize loss to follow-up for admitted and discharged patients in a setting where most patients do not have consistent ability to receive phone calls. Additionally, follow-up after three days was thought to be less reflective of outcomes related to acute care provided in the emergency unit. Ethics approval for the quality assurance database was obtained through the Institutional Review Board at Mbarara University of Science and Technology. Data was input during patient stays in the emergency unit by Global Emergency Care-trained research assistants present in the emergency unit. They used both Microsoft Excel (from 1 January 2010–23 March 2012) and Microsoft Access (24 March 2012–31 December 2019) databases for data input. Data was abstracted, cleaned, and analyzed by a single researcher (BR) using Stata 16.1 (StataCorp, College Station, TX).

### Data analysis

Secondary analysis was performed on prospectively collected data abstracted from the Karoli Lwanga Hospital emergency unit quality assurance database. This electronic database was generated from the paper charts of all consecutive patients presenting to the emergency unit from January 2010 until December 2019. Patients less than 18 years of age were excluded from analysis. Patient with atypical dispositions (referral, eloped, left against medical advice) did not receive standard follow-up and were also excluded from analysis. No discharged patients were referred to outside facilities. Suspected infections were defined by a complaint of fever, an objective fever, or a diagnosis consistent with infection (full list of diagnoses is available as a supporting file (S1 Appendix). Sepsis was defined as a patient with a suspected infection and a qSOFA score of one point or greater with one point each for: tachypnea (respiratory rate ≥ 22); altered mentation (defined as a Glasgow Coma Scale <15 or an AVPU score other than Alert); hypotension (systolic blood pressure ≤ 100). A cutoff of qSOFA ≥ 1 was chosen based on expert opinion regarding the utility of qSOFA in LMICs and to mitigate the risk of missing mental status data systematically biasing analysis towards under-representing sepsis. If more than one set of vitals was taken, the most abnormal vital was included in the qSOFA calculation. After consultation with clinicians, patients without recorded mental status were assumed to have a mental status of “Alert” for analysis, based on practice patterns of typically omitting recording mental status if it was normal. No imputation was otherwise performed for missing data. Hypotension in sepsis was defined as sepsis with a systolic blood pressure less than 90. Malaria was defined as either “smear-positive” (a positive thick/thin smear for Plasmodium falciparum), or “clinical” (patient received diagnosis or treatment of malaria prior to arrival and/or clinical suspicion was high enough despite a negative thick/thin smear). Demographics, vital signs, administration of anti-infectives (including antimalarial, antiviral, antifungal and/or antibiotic medicines) and intravenous fluids (normal saline and/or Ringer’s lactate solution), malaria testing, HIV status (previously known or tested in the emergency unit), and three-day mortality outcomes were analyzed for all patients. Non-parametric age data were compared using the Wilcoxon rank-sum test; continuous variables were compared using the t-test and one-way ANOVA; proportions were compared using Fisher’s exact test. A multi-variable logistic regression model to test the significance of associations between independent variables and mortality in septic patients was developed. Each candidate variable was tested for independent association with the primary outcome (death) and all variables with a p<0.2 were included in the multi-variable model. The only exception were the treatment variables (fluids, antibiotics, both) which were not tested for association but included based on accepted standards of sepsis care. Area Under Receiver Operating Characteristics Curve (AUROC), Hosmer-Lemeshow Goodness of Fit, and Brier score were all calculated for this model.

### Ethics approval and consent to participate

Ethics approval for the quality assurance emergency unit database was obtained through the Institutional Review Board at Mbarara University of Science and Technology and University of Massachusetts. Clinical care was provided independently of data collection, and with data being collected as part of ongoing quality assurance processes, individual consent was waived by the ethics committee. Subsequent analysis was performed on de-identified data.

### Patient and public involvement

The non-physician clinician training programme was originally developed in response to several years of clinical emergency medicine experience and ongoing health care staffing shortages in Uganda. The positive response of patients, staff and administrators at Karoli Lwanga Hospital to the training programme and their interest in improving patient care led to ongoing research and programme evaluation. Patients and the public were not involved in the design of the study however outcome measures are explicitly patient-oriented. Results will be disseminated through open access publication to allow local clinicians, administrators, policymakers and researchers to benefit.

## Results

**Fig 1:**
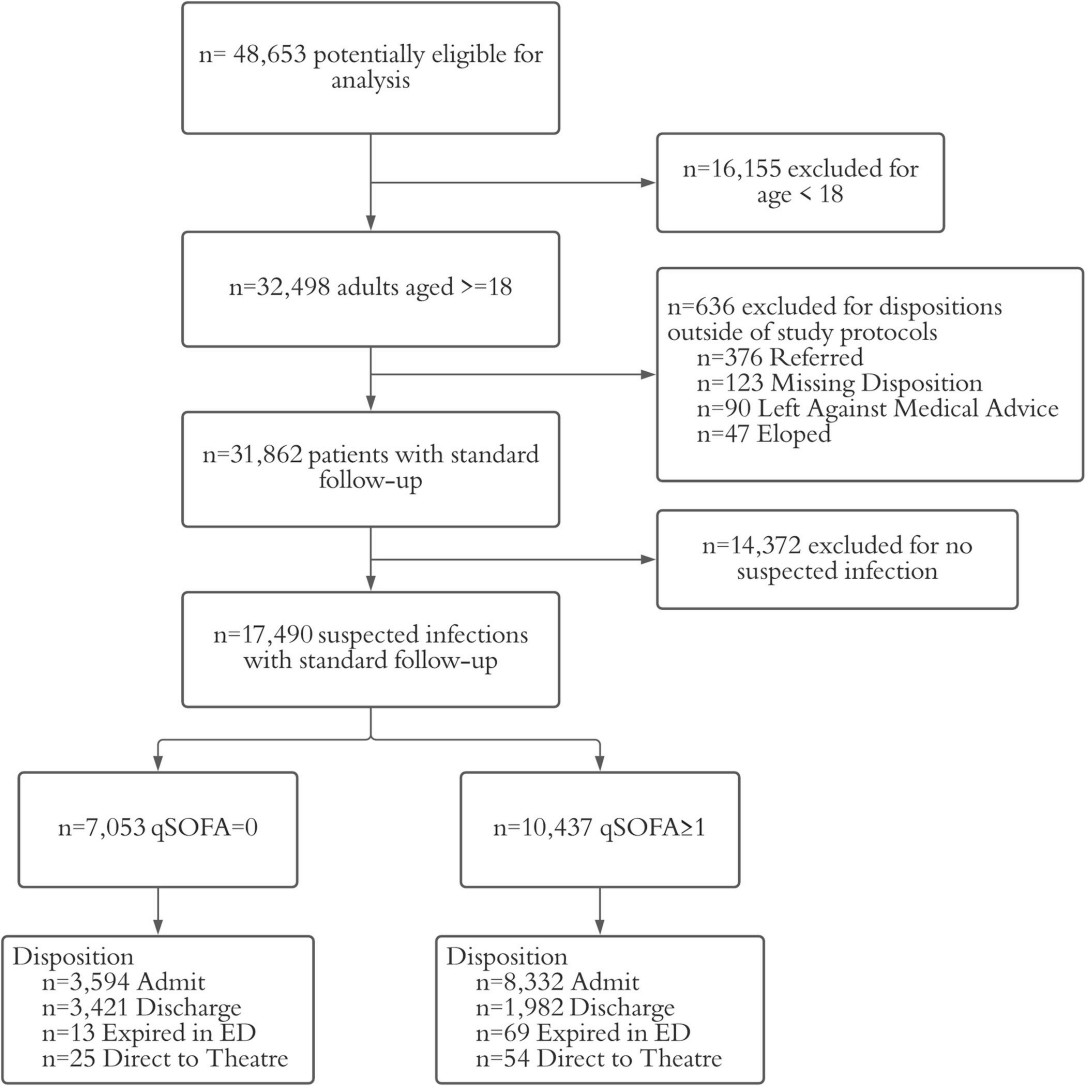
Patient visit inclusion and exclusion criteria.

Overall, there were 48,653 patient visits from 2010 to 2019 (Fig 1). Of these, 17,490 patients were included in the analysis who were aged 18 and older and had a suspected infection. In total, 10,437 patients were defined as having sepsis based on qSOFA scores of 1 or greater (7,114 qSOFA = 1, 3,190 qSOFA = 2, 133 qSOFA = 3). Loss to follow-up analysis was stratified by disposition as the emergency unit overall experienced fundamentally different rates of loss to follow-up for admitted (n = 702 of 11,927, 5.9%) and discharged patients (n = 2,748 of 5,403, 50.9%). When comparing septic and non-septic patients by disposition, admitted patients in both groups had similar loss to follow up (5.3% vs. 6.1%, p = 0.088), while discharged patients with sepsis were less likely to be lost to follow up than non-septic patients (n = 867 of 1,982, 43.7% vs. n = 1,881 of 3,421, 55.0%%, p<0.001). The overall mortality rate for combined septic and non-septic discharged patients with follow-up was extremely low with only 4 deaths (n = 4 of 2,655, 0.15%) over the 10 years of the study.

**Fig 2:**
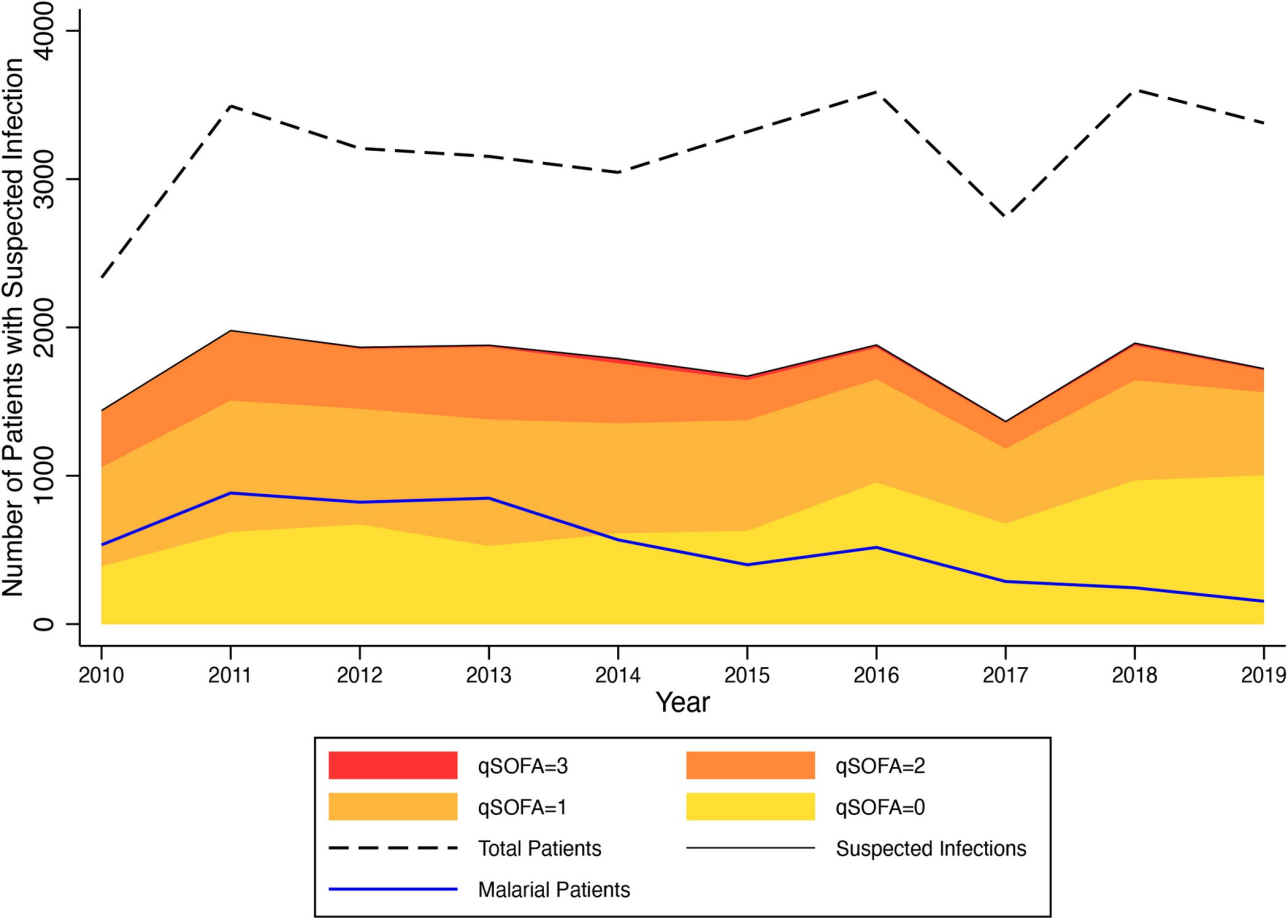
Annual emergency unit visits with suspected infection, qSOFA scores and malaria by year, 2010–2019.

The annual number of visits for all patients with suspected infections, their qSOFA scores and their malaria status are displayed in Fig 2. The proportion of all emergency unit visits for patients aged 18 years or older that met criteria for sepsis during the study period was 32.8% overall. The annual proportion of all visits with suspected infections decreased from 61.7% in 2010 to 51.0% in 2019, with the proportion of all visits who had sepsis decreased from 45.0% in 2010 to 21.3% in 2019. The annual proportion of all visits with malaria (combined “smear-positive” and “clinical”) decreased from 37.1% in 2010 to 8.9% in 2019. Non-malarial sepsis decreased from 27.3% of all visits in 2010 to 19.2% in 2019. Malarial sepsis had an even more pronounced decrease from its peak of 17.7% in 2013 to 2.1% in 2019. The proportions in Fig 2 were calculated using all emergency unit patient visits as a denominator (n = 31,856) to describe overall emergency unit trends. All subsequent analyses were restricted to patients with suspected infection (n = 17,490). The proportion of patients with suspected infections who had a qSOFA = 0 increased from 27.0% in 2010 to 58.3% in 2019; those with qSOFA = 1 decreased from 46.5% in 2010, to 32.5% in 2019; those with a qSOFA = 2 decreased from 26.5% in 2010 to 8.7% in 2019 and those with a qSOFA = 3 decreased from a peak of 1.8% in 2014 to 0.6% in 2019.

Characteristics of septic and non-septic patients with suspected infections were compared (Table 1).

**Table 1:** Characteristics of non-septic (qSOFA = 0) and septic (qSOFA≥1) emergency unit patients with suspected infections (N = 17,490).

	No Sepsis (qSOFA = 0)	Sepsis (qSOFA≥1)	p-Value
	n = 7,053	n = 10,437	
**Age, median (IQR)**	44 (26–70)	41 (27–64)	0.0001^†^
**Age Group**			
** 18–64 years old, total (%)**	4893 (67.4)	7835 (75.1)	<0.001
** 65+ years old, total (%)**	2111 (29.9)	2541 (24.4)	<0.001
**Female, total (%)**	3406 (48.3)	5724 (54.9)	<0.001
**Systolic Blood Pressure, mean (95% CI)**	125.2 (124.8–125.6)	107.6 (107.1–107.9)	<0.001^††^
**Heart Rate, mean (95% CI)**	88.1 (87.6–88.5)	98.8 (98.4–99.3)	<0.001^††^
**Respiratory Rate, mean (95% CI)**	18.7 (18.7–18.8)	26.1 (25.9–26.2)	<0.001^††^
**Oxygen Saturation, mean (95% CI)**	96.2 (96.0–96.3)	93.8 (93.7–93.9)	<0.001^††^
**qSOFA Criteria**			
** Respiratory rate ≥ 22 breaths per minute, n (%)**	0	7664 (73.4)	<0.001
** Systolic blood pressure ≤ 100 mmHg, n (%)**	0	2369 (22.7)	<0.001
** Altered mentation (GCS < 15 or AVP ≠ A), n (%)**	0	685 (6.6)	<0.001
**Co-existing Infections**			
** Malaria: Smear-Positive, n (%)**	897 (12.7)	2016 (19.3)	<0.001
** Malaria: Clinical, n (%)**	811 (11.5)	1536 (14.7)	<0.001
** HIV, n (%)**	423 (6.0)	1554 (14.9)	<0.001
**Clinician Impression, n (%)**			
** "Not Sick"**	4536 (64.3)	4168 (40.0)	<0.001
** "Sick"**	2429 (34.4)	5858 (56.1)	<0.001
** "Toxic"**	45 (0.6)	337 (3.2)	<0.001
**Disposition, n (%)**			
** Admitted**	3594 (51.0)	8332 (79.8)	<0.001
** Discharged**	3421 (48.5)	1982 (19.0)	<0.001
** Expired in ED**	13 (0.2)	70 (0.7)	<0.001
** Operating Theater**	25 (0.4)	54 (0.5)	0.14

^†^ Wilcoxon rank-sum used as test of significance

^††^ T-test used as test of significance

All others use Fisher’s exact test as test of significance

There were significant differences between the groups, with septic patients being younger, more likely to be female, more likely to have abnormalities in all vital signs (blood pressure, heart rate, respiratory rate, oxygen saturation, mental status), more likely to be coinfected both with HIV and malaria, more likely to have a clinician impression of “Serious” or “Critical” illness, and more likely both to be admitted to the hospital and to die in the emergency unit. Levels of missing data were generally low: age (n = 110, 0.63%), gender (n = 12, 0.07%), blood pressure (n = 681, 3.89%), heart rate (n = 515, 2.94%), respiratory rate (n = 1,460, 8.35%), oxygen saturation (n = 1,338, 7.65%), and clinician impression of illness severity (n = 118, 0.67%). Mental status was an exception with a high level of missing data (n = 10,720, 62.7%) with AVPU being most commonly recorded (n = 6,174, 35.3%), Glasgow Coma Score being less commonly recorded (n = 252, 1.4%) and both being recorded rarely (n = 350, 2.0%). For completeness and sensitivity analysis, all analysis described below was performed using qSOFA ≥2 as the cutoff for sepsis and is attached to this manuscript as supporting files (S1–S3 Tables, S1–S5 Figs, S1 Text).

The mortality and interventions in patients stratified by qSOFA score are displayed in Table 2 below. Interventions were incompletely captured for the 3,942 patients in the original Excel database from 2010–2012, therefore analysis of interventions in sepsis over time was restricted to the 13,548 patients recorded in the Access database from 2012–2019. Increasing qSOFA scores were associated with monotonically increasing rates of death and receipt of “both fluids and anti-infectives” and monotonically decreasing rates of receiving “neither fluids nor anti-infectives”. The dichotomous splitting of patients into “No Sepsis” and “Sepsis” using the cutoff of qSOFA≥1 shows the same clinically and statistically significant increases and decreases.

**Table 2:** Interventions and mortality for patients 2012–2019 stratified by qSOFA score (n = 13,548).

	qSOFA Score					Dichotomous qSOFA Score		
	Zero	One	Two	Three	p-Value	No Sepsis (= 0)	Sepsis (≥ 1)	p-Value
**Total Cases, n**	5855	5331	2229	133	n/a	5855	7693	n/a
**Total Deaths, n**	65	215	189	27	n/a	280	216	n/a
**Crude Mortality Rate, % [95% CI]**	1.1 [0.9–1.4]	4.0 [3.5–4.6]	8.5 [7.4–9.7]	20.3 [13.8–28.1]	<0.001^†^	1.1 [0.8–1.4]	5.6 [5.1–6.1]	<0.001^††^
**Proportion receiving interventions, (%)**								
** **Neither fluids nor anti-infectives	3565 (60.9)	2168 (40.7)	505 (22.7)	18 (13.5)	0.08^†^	3565 (60.9)	2691 (35.0)	<0.001**
** **Fluids only	837 (14.3)	1092 (20.5)	528 (23.7)	29 (21.8)	0.009^†^	837 (14.3)	1649 (21.4)	<0.001**
** **Anti-infectives only*	794 (13.6)	966 (18.1)	380 (17.1)	16 (12.0)	0.004^†^	794 (13.6)	1362 (17.7)	<0.001**
** **Both fluids and anti-infectives	659 (11.3)	1105 (20.7)	816 (36.6)	70 (52.6)	0.06^†^	659 (11.3)	1991 (25.9)	<0.001**

^†^ ANOVA used as test of significance

^††^ T-test used as test of significance

* Anti-infectives include antibiotics, antivirals and/or antimalarials

** Fisher’s exact test used as test of significance

**Fig 3:**
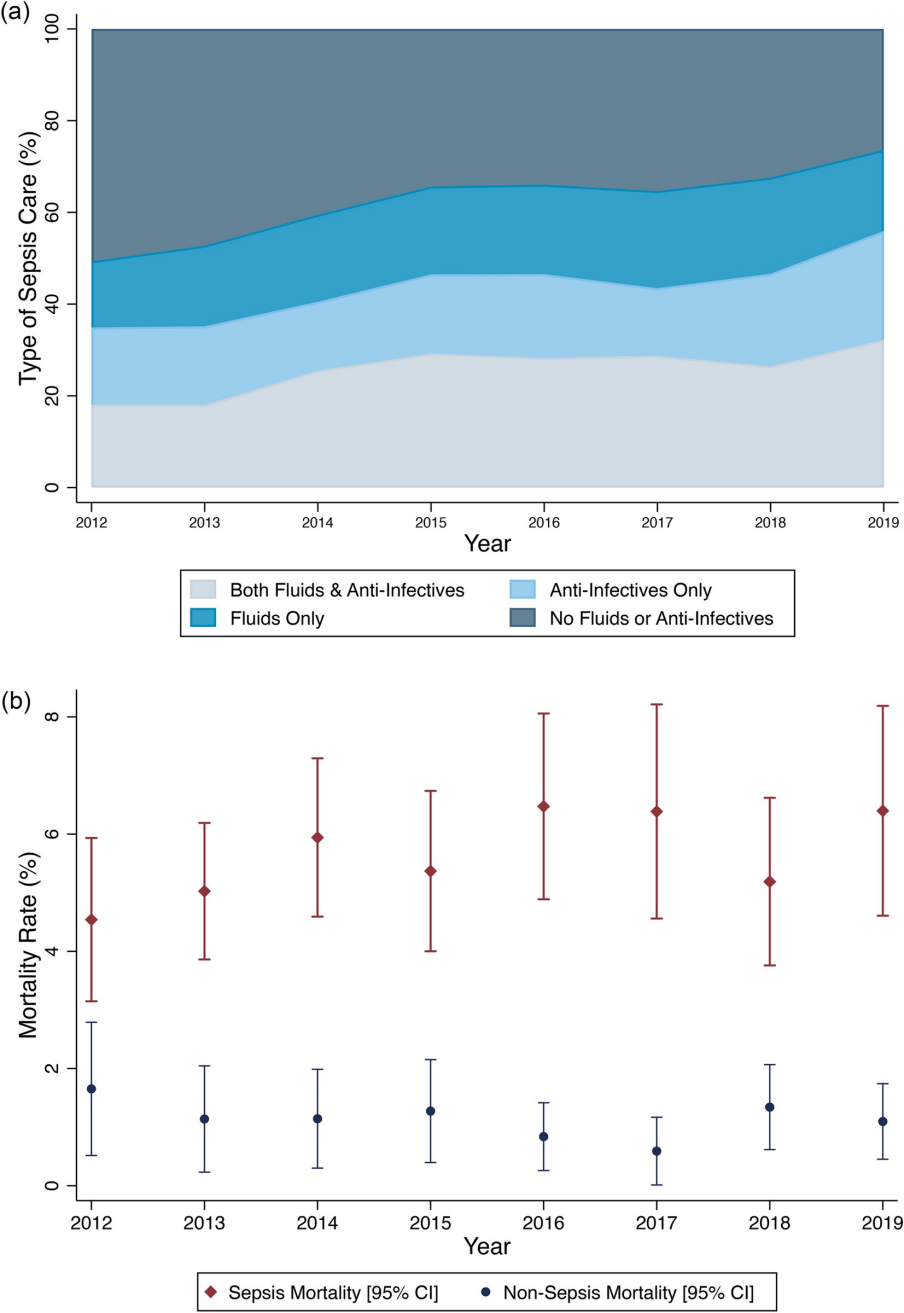
Trends in sepsis management and sepsis mortality, 2012–2019. Top graph shows proportion of sepsis management over time. Bottom graph shows sepsis mortality over time compared with non-sepsis mortality.

Fig 3 displays the annual trends in sepsis management and mortality from 2012–2019. The rates of receiving both fluids and anti-infectives increased (21.2% in 2012 to 32.0% in 2019), while the rates of receiving *neither* fluids *nor* anti-infectives in the emergency unit decreased (41.7% in 2012 to 19.6% in 2019) (Fig 3A, top). Over that same time period, changes in mortality were not significant for patient with sepsis (4.5% in 2012 to 6.4% in 2019, p = 0.50) or patients without sepsis (2.7% in 2012 to 2.2% in 2019, p = 0.75) (Fig 3B, bottom). Mortality data for 2010 and 2011 are not included in Fig 3B for consistency with Fig 3A and are as follows: in 2010, non-sepsis mortality was 1.8% [95% CI 0.5–3.1] and sepsis mortality was 5.4% [95% CI 4.1–6.8]; in 2011 non-sepsis mortality was 1.3% [95%CI 0.4–2.2] and sepsis mortality was 4.6% [95%CI 3.5–5.8].

**Fig 4:**
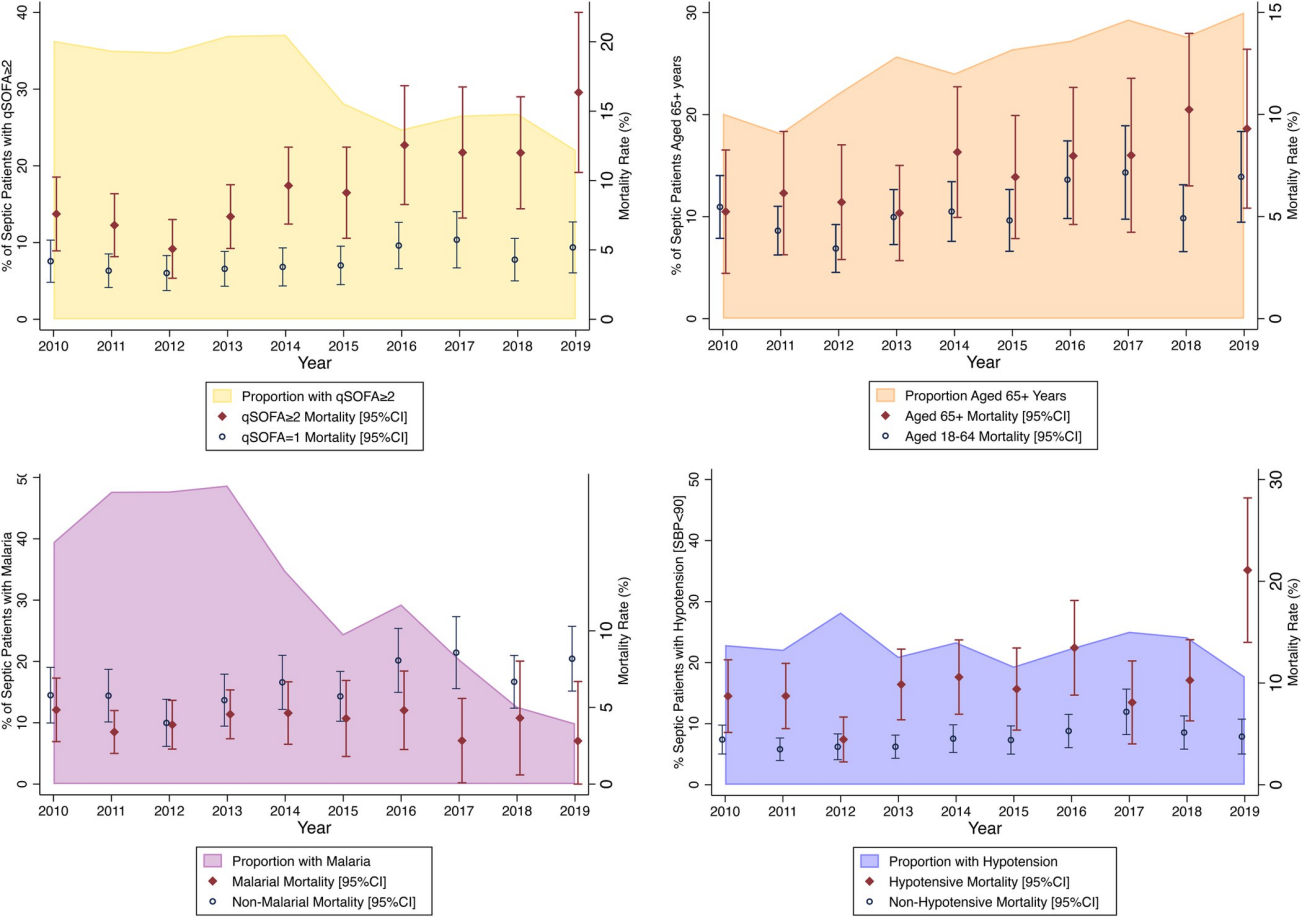
Proportions and comparative mortality for sub-populations of septic (qSOFA≥1) patients, 2010–2019.

Fig 4 displays trends in prevalence and associated mortality for sub-groups of septic patients by year from 2010–2019. Across programme years, septic patients are more likely to be elderly and to have qSOFA≥2 (both higher mortality sub-groups), less likely to have malaria (a lower mortality sub-group) and equally likely to have hypotension (a higher mortality sub-group).

Clockwise description of annual trend graphs from top left: proportion of patients with qSOFA≥2 sepsis overlaid with mortality for patients with qSOFA≥2 and qSOFA = 1; proportion of patients aged 65 and over overlaid with mortality between subgroups age 65 and over and age 18–64; proportion of patients presenting with hypotension overlaid with mortality between hypotensive and non-hypotensive subgroups; proportion of patients with malaria overlaid with malarial and non-malarial mortality.

Looking at malarial sepsis only (n = 2339), there were 86 deaths (4.3% mortality rate) in the 2016 cases from 2012–2019. Further analysis of this data over time was limited by the precipitous drop in the proportion of patients with malaria and associated deaths culminating in the years 2017–2019 having 60, 29 and 21 cases and zero, two and two deaths respectively. With the rapid reduction in annual malaria cases and deaths, and with expert opinion suggesting that fluids may be more dangerous in malarial sepsis, logistic regression for malarial sepsis mortality is not included as primary analysis in this manuscript but is available as a supporting file (S4 Table) [28, 29].

Analysis of non-malarial sepsis (n = 5354) used a multiple variable logistic regression model for mortality (Table 3). In this model, the odds ratio (OR) of death was significantly associated with increased mortality for almost all included variables. Exceptions include a significant protective effect seen with fever and with female gender, and a failure to meet statistical significance with altered mental status. The largest OR was associated with clinical condition upon arrival (“Sick” and “Toxic”). The p-value for the Hosmer-Lemeshow goodness of fit test for the model was 0.26, the Brier score was 0.062, and the AUROC was 0.82 (95%CI 0.80–0.85).

**Table 3:** Logistic regression model of mortality in septic (qSOFA≥1) patients without malaria: 2012–2019 (N = 5,323).

	OR	95% CI			p-Value
Age					
Additional Year (above 18)	1.01	1.00	-	1.01	0.006
HIV					
Negative	REF				
Positive	1.63	1.2	-	2.2	0.002
Gender					
M	REF				
F	0.49	0.4	-	0.6	<0.001
Respiratory Status					
Normal Rate + No Hypoxia	REF				
Normal Rate + Hypoxia (SpO2<92%)	1.35	0.9	-	2.0	0.129
Tachypnea (≥22 bpm) + No Hypoxia	1.69	1.0	-	3.0	0.069
Tachypnea (≥22 bpm) + Hypoxia (SpO2<92%)	4.57	3.1	-	6.7	<0.001
Heart Rate					
Normal	REF				
Tachycardic (≥100 bpm)	1.35	1.0	-	1.7	0.023
Temperature					
Hypothermic (≤ 35.5°C)	1.78	1.3	-	2.4	<0.001
Normal	REF				
Febrile (≥ 37.5°C)	0.58	0.4	-	0.8	0.002
Blood Pressure					
Not Hypotensive	REF				
Hypotensive (SBP<100)	1.63	1.3	-	2.1	<0.001
Mental Status					
Normal/Not Recorded	REF				
Altered	1.36	1.0	-	1.9	0.081
Clinical Impression					
"Not Sick"	REF				
"Sick"	3.0	2.1	-	4.5	<0.001
"Toxic"	17.6	10.8	-	28.5	<0.001

**Fig 5:**
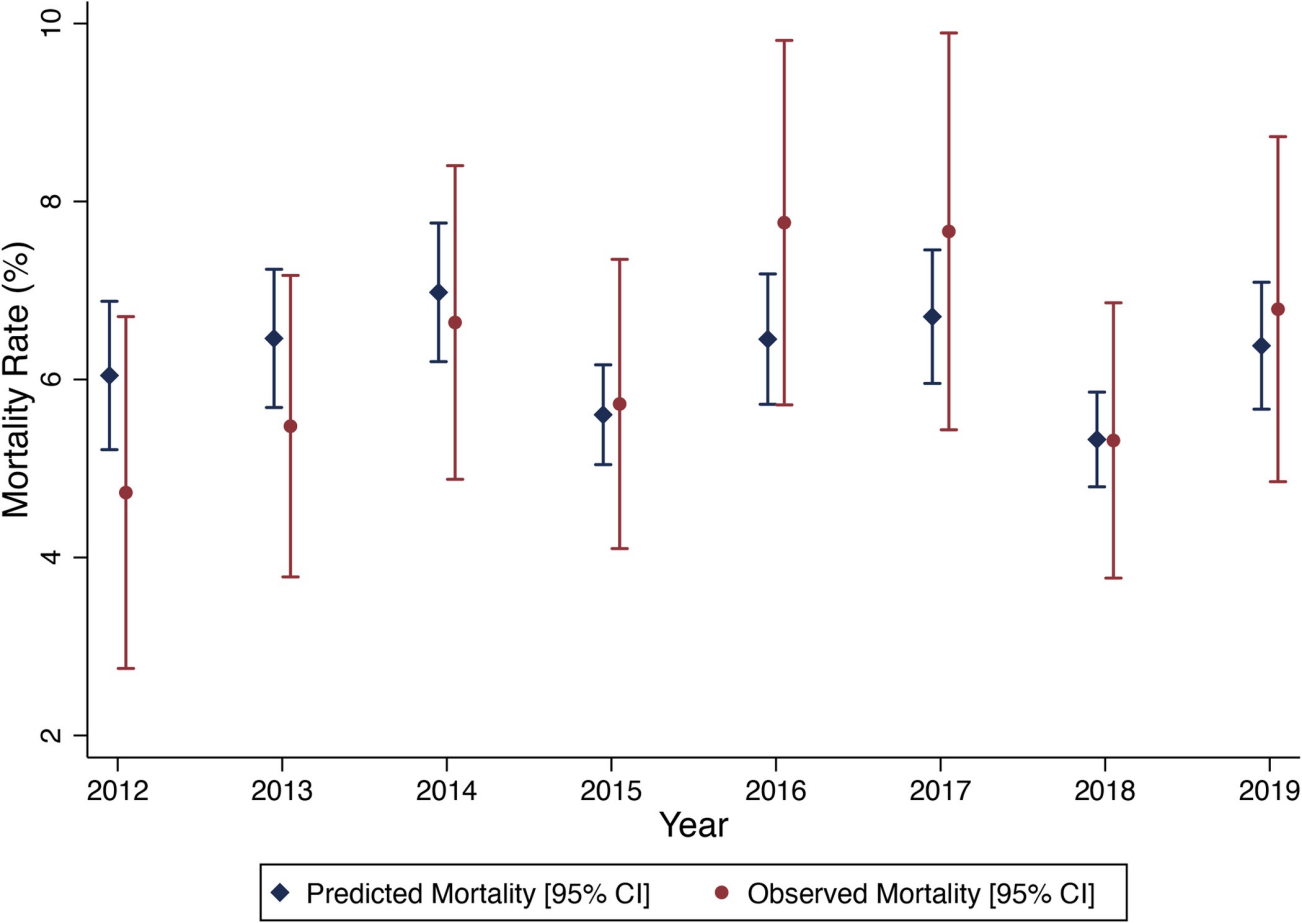
Annual predicted and observed mortality in septic (qSOFA≥1) patients without malaria, 2012–2019.

Using all the variables in the above logistic regression model (Table 3), coefficients were generated representing expected mortality for each patient. These coefficients were combined by year to generate annual predicated mortality and were plotted against observed mortality across programme years (Fig 5).

In all years, there is no significant difference between predicted and observed mortality and no clear trend exists for observed mortality being greater or less than predicted mortality.

For analysis of the mortality impact of sepsis treatment in the emergency unit (no treatment in the emergency unit, fluids alone, antibiotics alone, or both fluids and antibiotics), this categorical was added to the logistic regression model (Table 3) to assess the independent association between treatments and mortality septic patients without malaria. The marginal contribution of this variable to the model overall was used to calculate relative risks (RR) associated with each category of treatment. Treatment of sepsis with “both fluids and antibiotics” (RR = 1.55, 95%CI 1.10–2.00) was associated with a significantly *increased* RR of death as compared to “no treatment in the emergency unit” after controlling for other variables in the logistic regression model above. There was no statistically significant increase or decrease in mortality for treatment with “fluids alone” (RR = 1.24, 95%CI 0.83–1.66) or “antibiotics alone” (RR = 1.19, 95%CI 0.81–1.56).

## Discussion

The analysis presented above describes a decade of Ugandan emergency unit experience with sepsis management and outcomes. The 10,437 septic patients (including 7,114 with qSOFA = 1 and 3,323 with qSOFA≥2) in this analysis represent the largest study of sepsis outcomes in SSA to date. In fact, this cohort represents more patients than the 2800 patients in all other published studies of sepsis outcomes in SSA combined [30]. The number of subjects alone makes the study noteworthy as does the longitudinal nature of the data providing insight into the changing landscape of sepsis and emergency care in Uganda over the last decade. Additionally, this analysis provides strong evidence that an emergency care training programme for non-physician clinicians can produce and sustain improvements in care quality as defined by adherence to expert recommendations at the time.

Sepsis was a substantial burden in this Ugandan emergency unit over a decade with 32.8% of all adult emergency unit visits meeting criteria for sepsis. However, stratifying the proportion of patients with sepsis by year showed a marked decrease from 45.0% of all patient visits in 2010 to 21.3% in 2019. Though both overall sepsis and non-malarial sepsis decreased over time, the drop in the proportion of patients with malarial sepsis was profound: 17.2% in 2010 to 2.1% in 2019. These findings are in accord with the observed decrease in malaria as a cause of death and disability in Uganda over the period of 2007–2017 in the Global Burden of Diseases, Injuries and Risk Factors (GBD) survey [2]. The National Malaria Control Programme in Uganda was active during the study period, and employed a combination of control measures including long-lasting insecticidal nets, indoor residual spraying, and intermittent preventive treatment for malaria during pregnancy. The success of this program is the likely cause of the observed reduction in emergency unit burden [31]. The sharp downward trend in non-malarial sepsis and sepsis overall seen here has also been observed during the study period in national and regional level analyses of GBD data [2, 32]. While GBD data is not specific to the emergency unit, the few emergency medicine-focused studies from SSA show similar downward trends in sepsis incidence [33]. The ultimate causes of this trend are likely tied to overall economic and health systems development in Uganda and throughout SSA in general, but detailed analysis of the impact of those forces on emergency unit malaria incidence and mortality is outside the scope of this manuscript.

The data presented in Table 2 showed that qSOFA performed well in classifying patients with higher and lower mortality rates in line with other research suggesting the value of qSOFA in SSA [10]. Using the cutoff of qSOFA≥1 to define sepsis clearly identified patients at increased risk of death, while simultaneously addressing concerns that missing mental status data might bias the study away from identifying septic patients. The alternative analysis using a qSOFA≥2 as the cutoff for sepsis produced notably similar results including model performance and a significant marginal increase in mortality associated with treatment with “both fluids and antibiotics” in the emergency unit (S1–S3 Tables, S1–S5 Figs, S1 Text). While detailed comparison between those two scoring cutoffs is not the focus of the manuscript, these findings support the overall evidence base that not only is qSOFA applicable in low-resource settings but that using a qSOFA≥1 to define sepsis risk may be more appropriate in SSA emergency unit settings [10, 11]. Overall, this represents the first study to the authors’ knowledge that demonstrates the potential utility of qSOFA by non-physician clinicians. Further studies would be needed to formally validate the utility of qSOFA by non-physician clinicians.

Non-physician clinician sepsis care quality also improved significantly during the study period (Fig 3A). The non-physician clinician training employed in the study setting promotes ongoing practice improvement and has focused on increasing the rates of early resuscitation of septic patients in accordance with regional guidelines for emergency care [24]. The rates of fluid and antibiotic administration provided by non-physician clinicians compared favorably to the standard of care provided by admitting medical officers in other Ugandan hospitals during the same time period [9]. Some authors have suggested that improved training of nurses, paramedical assistants, and other non-physician clinicians could significantly improve sepsis identification and management and can be done at low cost [6, 34]. Taking sepsis care as a reasonable proxy for emergency care in general, the findings of this manuscript have clear policy implications: emergency medicine training programmes for non-physician clinicians produce a workforce capable of delivering quality care, as defined by meeting published care guidelines. Such programmes may address both emergency care staffing shortages in SSA and help meet the pressing global development goals of improving quality of care [35].

Despite the increasing quality of care, the observed mortality for septic patients did not improve significantly over time (Fig 3). Given the global trends towards reduced sepsis incidence and mortality cited above, this was somewhat surprising. Notably, the characteristics of the septic population did change over the same period (Fig 4). The national-level trends in Uganda of an aging population, increasing rates of non-communicable diseases and comorbidities and decreasing rates of malaria prevalence contributes means that septic patients in 2019 had quite different characteristics than in 2010. Additionally, “self-triage” or the decisions made by patients about where to seek emergency care for themselves or their family, likely also played a role during the study. The local providers and patients have given feedback to the development programme for many years that the local community has viewed the emergency unit positively since it was established in 2008, likely leading increasingly sick patients to preferentially seek care there.

The multi-variable logistic regression model was generated to control for the confounding effects of these trends and was found to be well-calibrated and have adequate ability to discriminate between patients at higher and lower risk for death. One variable not commonly included in mortality models–but which independently predicted increased mortality in this model–was the initial “clinical impression” of patients upon their arrival to the emergency unit. The training programme has long stressed that the clinical skill of rapidly identifying both critically ill and generally well-appearing patients is a cornerstone of emergency care. Every non-clinician trained by the programme was taught to categorize patients as “not sick”, “sick” or “toxic” early in their evaluation and prioritize interventions based on this assessment. The strongly significant independent association of this variable with mortality (“Sick” OR = 3.0; “Toxic” OR = 17.6) even when controlling for comorbidities, age and vital sign abnormalities suggests that this type of clinical assessment has utility in addition to other objective data in the emergency unit. This skill is central to emergency medicine physician training and the ability of emergency care-trained, non-physician clinicians to utilize it to reliably identify patients at higher risk for short-term mortality contributes to the overall argument for their clinical capacity to evaluate and provide independent emergency care for undifferentiated septic patients.

Despite the clearly increasing adherence to quality care guidelines across time (Fig 3A) there was no trend towards reducing crude sepsis mortality. Additionally, after plotting the predicted mortality generated from the coefficients of the logistic regression model, there was no trend towards a reduction in observed versus predicted mortality (Fig 5). Trying to understand the interplay between sepsis treatment and mortality in SSA is important as optimal sepsis care in SSA remains controversial with a recent review of sepsis guidelines in SSA citing multiple studies showing harm from fluids challenging the widely accepted role of fluids in sepsis resuscitation [18, 20, 36, 37].

Retrospective analysis such as that used in this study is challenged by one of the first principles of emergency medicine: the most critically ill patients with the highest mortality rates receive the most interventions. Table 2 provides supporting data for the existence of this care pattern by demonstrating significant associations between increasing interventions, increasing mortality and increasing qSOFA scores. This practice pattern ultimately creates substantial *confounding by indication* in associating interventions with mortality without clear causation. The logistic regression model for mortality was developed to control for confounding by indication by including not only objective data (e.g., vital signs) but also subjective data (e.g., clinical impression). It was hoped that the inclusion of subjective evaluation would control for the recognition of critical illness by the providers and their associated decisions to escalate interventions. Given concerns about the possible harm of fluid in sepsis, it was notable that administration of “fluids alone” (RR = 1.24, 95%CI 0.83–1.66) was not significantly associated with increased RR of death. However, the administration of “both fluids and antibiotics” had a clinically and statistically significant association with increased RR of death (RR = 1.54, 95%CI 1.02–2.69). This same significant effect was seen in the sensitivity analysis using a sepsis cutoff of qSOFA≥2 (S3 Table and S1 Text). Since no literature published to date suggests that antibiotics increase mortality in sepsis in any setting this significant association functions as a falsification test arguing that the model was unable to adequately control for confounding. This finding in turn argues against drawing any conclusions regarding causative associations between treatments for sepsis and mortality in this model.

Different analytic approaches may provide additional answers but, given that this manuscript includes a decade of data and more septic patients than exist in all other studies of sepsis outcomes in SSA combined, retrospective analysis may be inadequate to define optimal sepsis care in Uganda or SSA due to this overwhelming confounding by indication. As health systems and emergency care rapidly develop in Uganda and SSA more generally, they do so without the necessary evidence base to guide optimal sepsis care. Controlled, prospective, randomized clinical trials are needed to isolate the mortality impact of individual sepsis treatments and/or care bundles for emergency unit patients in these settings.

## Limitations

There are several limitations for this study. The first limitations are those of the registry database. Loss to follow-up for discharged patients was high, despite rigorous methods that included calling patients every day for seven consecutive days following discharge. However, the large number of patients that did not have phones or answer their phones limited response rates. Despite mortality being exceedingly low at 0.15% over 10 years of discharged patients, this low response rate likely resulted in underestimating discharged mortality. Multiple imputation of mortality data to control for this was considered but deemed methodologically unsound. Secondly, the registry data are limited to a single center. The regional specificity of causes and management of sepsis may limit the generalizability of the findings above. Mortality reported in this study was lower overall than in similar settings. This may be due to larger trends in mortality in the region but also likely reflects limitations of the emergency unit registry data which only recorded vital status at three days and therefore may underestimate longer-term measures such as total in-hospital mortality. Unique patient visits were recorded and reported instead of unique patients which may impact the proportion of patients with sepsis overall. As discussed above, logistic regression models were likely limited in their ability to control for confounding by indication. Limitations in data prevented inclusion weight-based dosing of fluids and appropriateness of anti-infectives as variables in analysis. Future randomized prospective studies may benefit from looking specifically at sepsis outcomes related to types of anti-infectives and volume of fluids administered. Missing data regarding mental status (omitted in over 60% of charts) may have systematically under-recognized sepsis based on qSOFA and was addressed by using qSOFA≥1 as the cutoff for sepsis.

## Conclusions

Data for over 10,000 septic patients from 2010–2019 at a single rural Ugandan emergency unit staffed by non-physician clinicians were included in retrospective analysis and represent the largest study of sepsis outcomes in SSA published to date. The annual proportions of patients with sepsis decreased over time, and the non-physician clinicians providing emergency care significantly improved their adherence to sepsis guidelines. These findings provide insight into how sepsis in Ugandan emergency medicine reflects sepsis trends at national and regional levels and demonstrate that non-physician clinicians are both capable of delivering high-quality emergency care in SSA and represent a possible solution to ongoing healthcare staffing shortages. As the nature of patients seeking emergency sepsis care changed over time the expected mortality rates increased but, despite improvements in care quality, the observed mortality rates also increased. Ultimately, logistic regression models were unable to control for confounding and isolate the mortality impact of fluids and/or antibiotics in sepsis. With retrospective analysis unable to address ongoing concerns about the safety of fluids in sepsis in SSA and emergency medicine developing rapidly in both Uganda and SSA, randomized controlled trials are urgently needed to define and guide optimal sepsis management.

## Supporting information

S1 Fig Patient visit inclusion and exclusion criteria, analysis of qSOFA <2 and qSOFA≥2. (TIF)

S2 Fig Annual emergency unit visits with suspected infection, qSOFA scores and malaria by year, 2010–2019. By virtue of stratification by qSOFA score, these data are the same as Fig 2.
(TIF)

S3 Fig Trends in sepsis management and sepsis mortality, 2012–2019, qSOFA≥2. Top graph shows proportion of sepsis management over time. Bottom graph shows sepsis mortality over time compared with non-sepsis mortality.
(TIF)

S4 Fig Proportions and comparative mortality for sub-populations of septic (qSOFA≥2) patients, 2010–2019. (TIF)

S5 Fig Annual predicted and observed mortality in septic (qSOFA≥2) patients without malaria, 2012–2019. (TIF)

S1 Table Characteristics of non-septic (qSOFA<2) and septic (qSOFA≥2) emergency unit patients with suspected infections (N = 17,490). (DOCX)

S2 Table Interventions and mortality for patients 2012–2019 stratified by qSOFA score (n = 13,549). Dichotomous analysis was done for qSOFA<2 and qSOFA≥2.
(DOCX)

S3 Table Logistic regression model of mortality in septic (qSOFA≥2) patients without malaria: 2012–2019 (N = 1,621). (DOCX)

S4 Table Logistic regression model of mortality in septic (qSOFA≥1) patients with malaria: 2012–2019 (N = 2,332). (DOCX)

S1 Text Supplemental logistic regression analysis of sepsis treatment association with mortality in nonmalarial septic patients. (DOCX)

S1 Appendix List of diagnoses consistent with infection. (DOCX)

## List of Abbreviations

CI: confidence interval
ECP: emergency care practitioner
LMICs: low- and middle-income countries
OR: odds ratio
qSOFA: quick Sequential Organ Failure Assessment
RR: relative risk
SSA: Sub-Saharan Africa
