# Supplementary figures and images for: Emergency care of sepsis in sub-Saharan Africa: Mortality and non-physician clinician management of sepsis in rural Uganda from 2010 to 2019
Source: PLoS One. 2022 May 11;17(5):e0264517. doi: 10.1371/journal.pone.0264517 (PMC9094533; doi:10.1371/journal.pone.0264517)

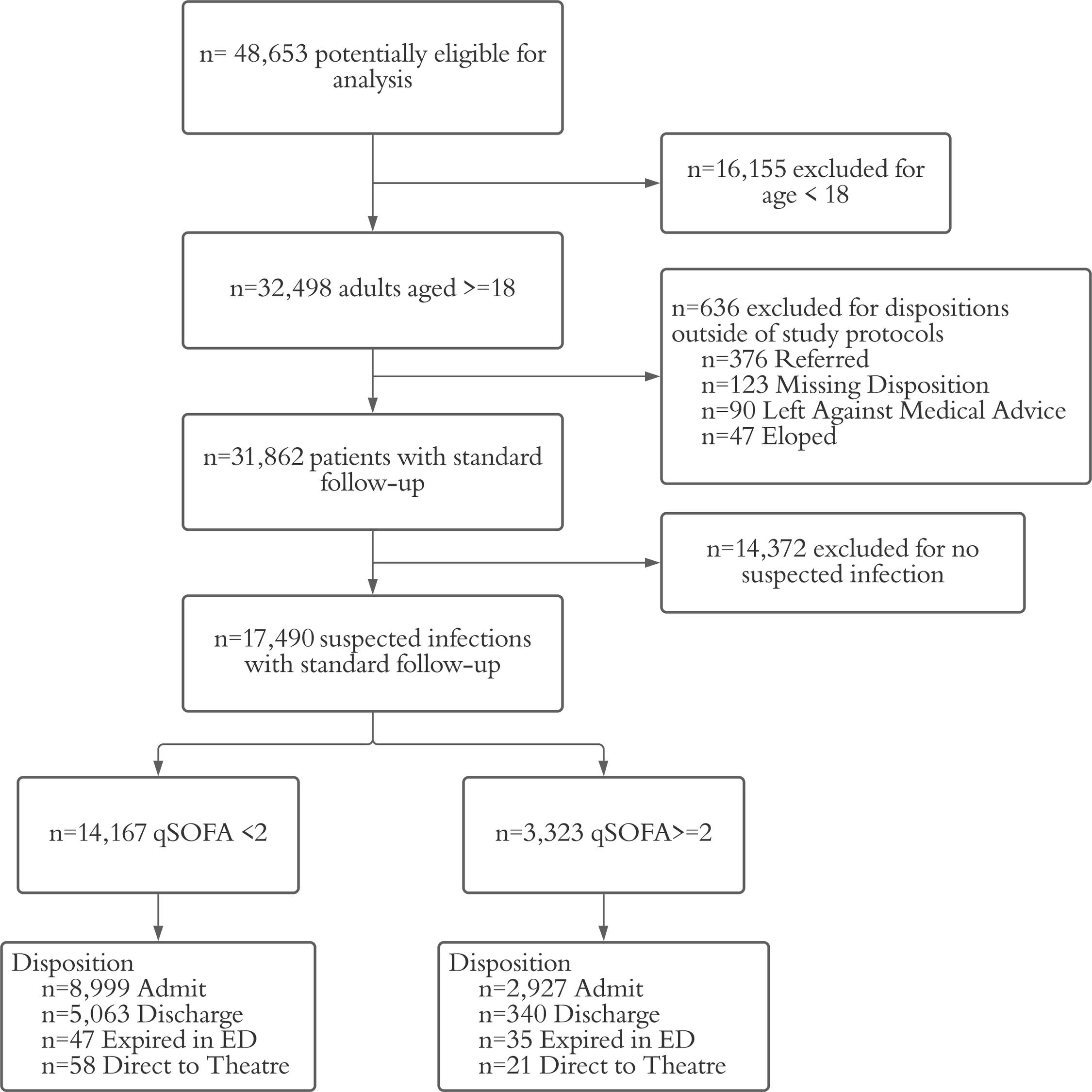

Supplement: S1 Fig — (TIF) [file pone.0264517.s001.tif]

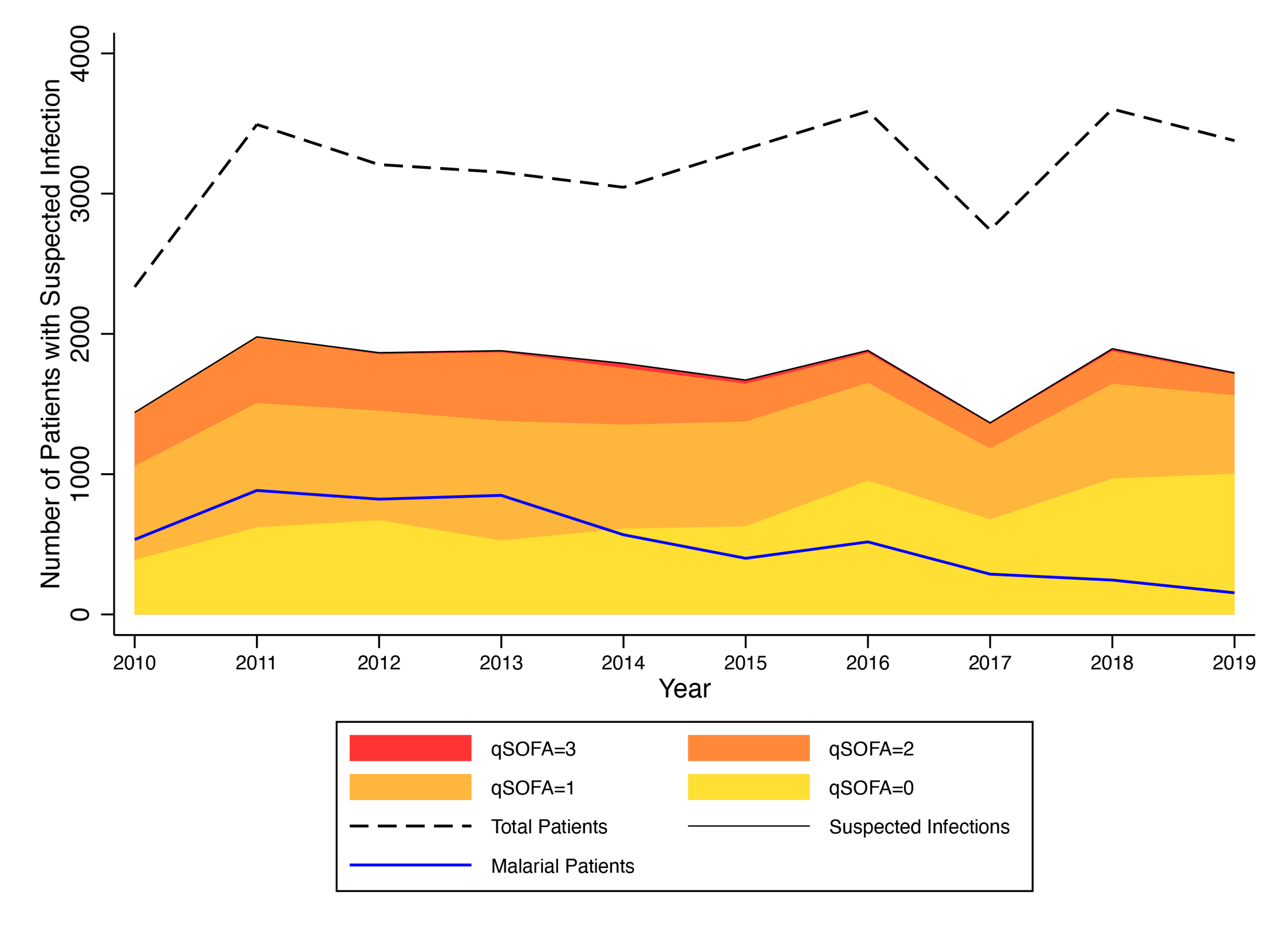

Supplement: S2 Fig — By virtue of stratification by qSOFA score, these data are the same as Fig 2. (TIF) [file pone.0264517.s002.tif]

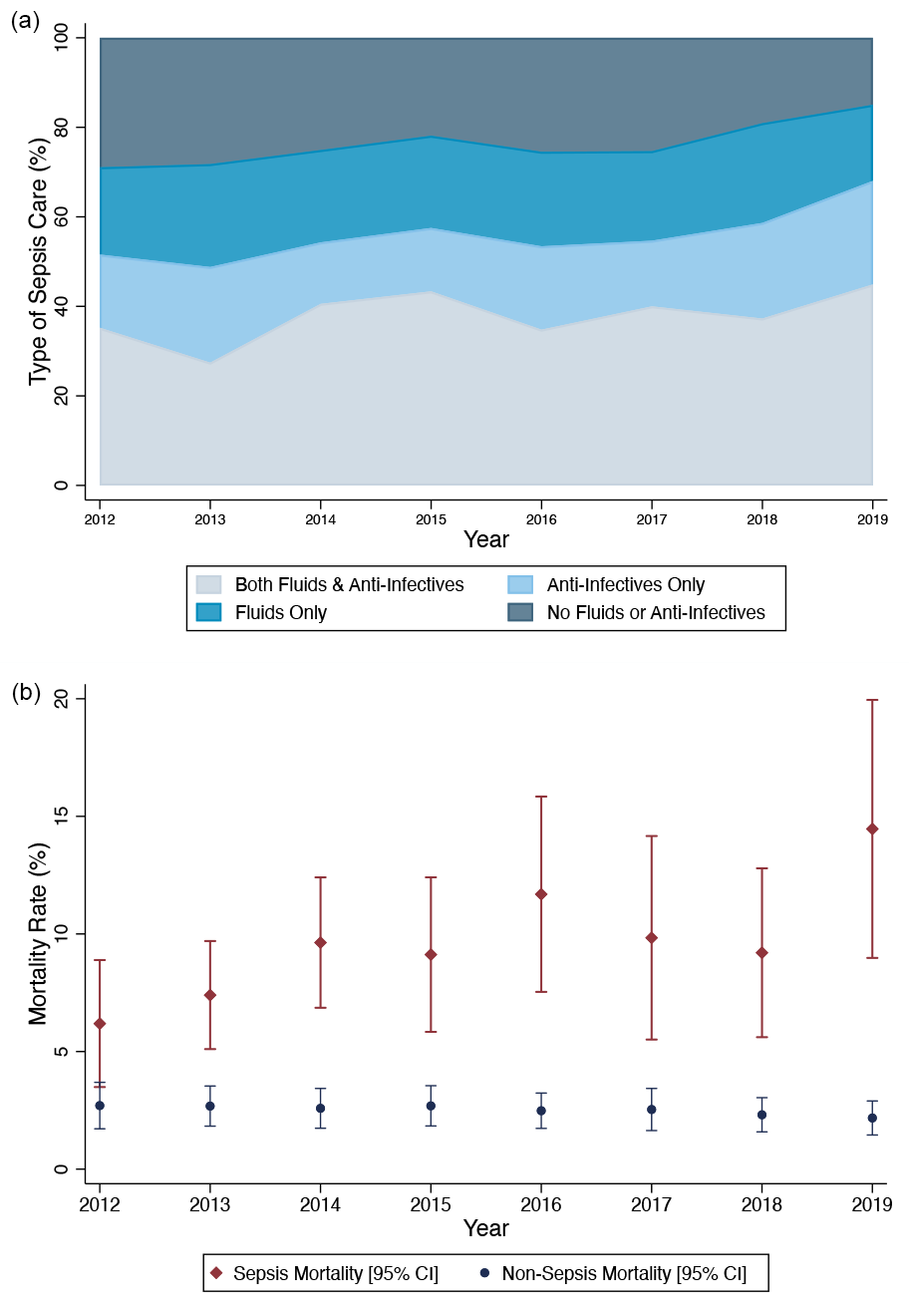

Supplement: S3 Fig — Top graph shows proportion of sepsis management over time. Bottom graph shows sepsis mortality over time compared with non-sepsis mortality. (TIF) [file pone.0264517.s003.tif]

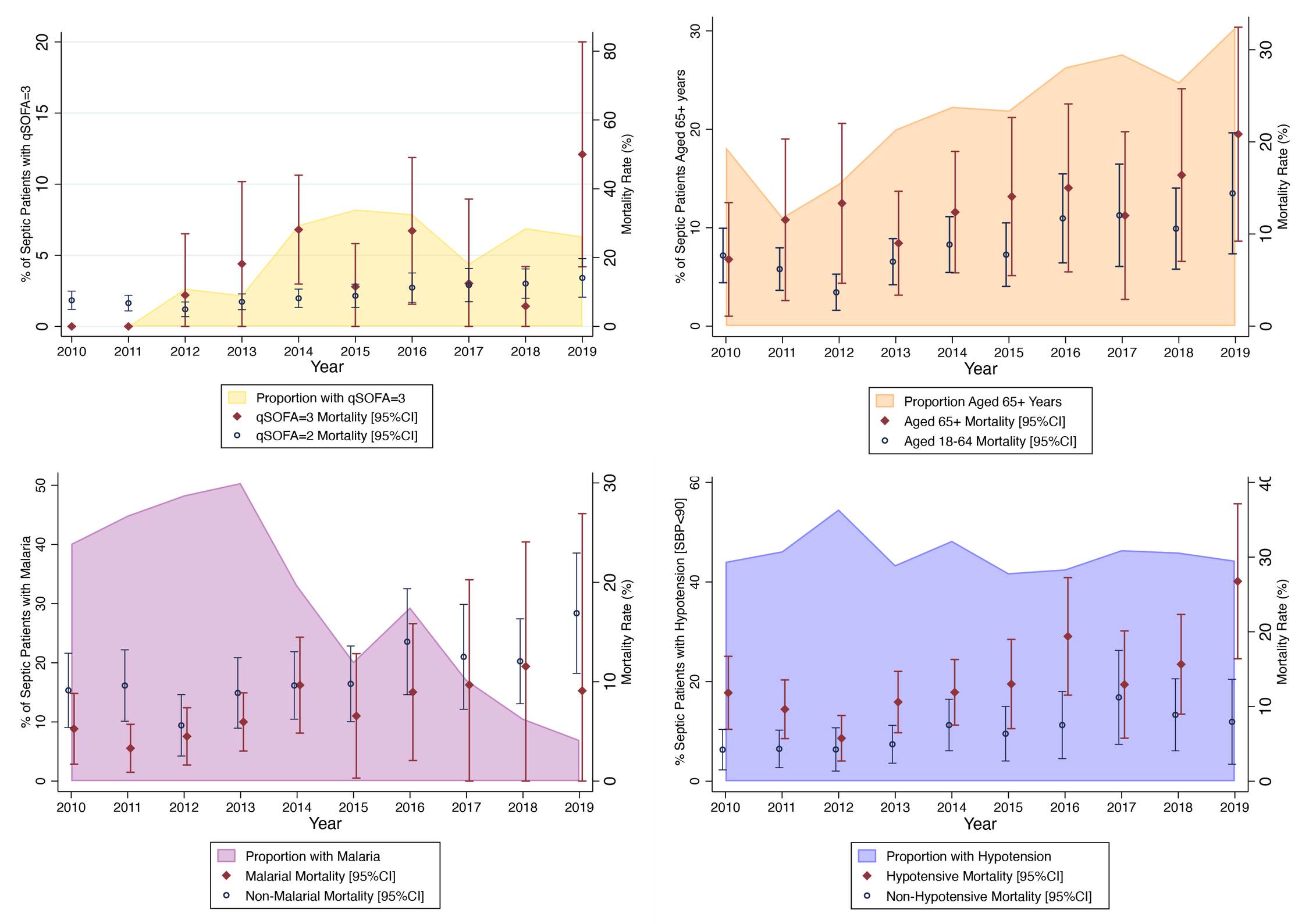

Supplement: S4 Fig — (TIF) [file pone.0264517.s004.tif]

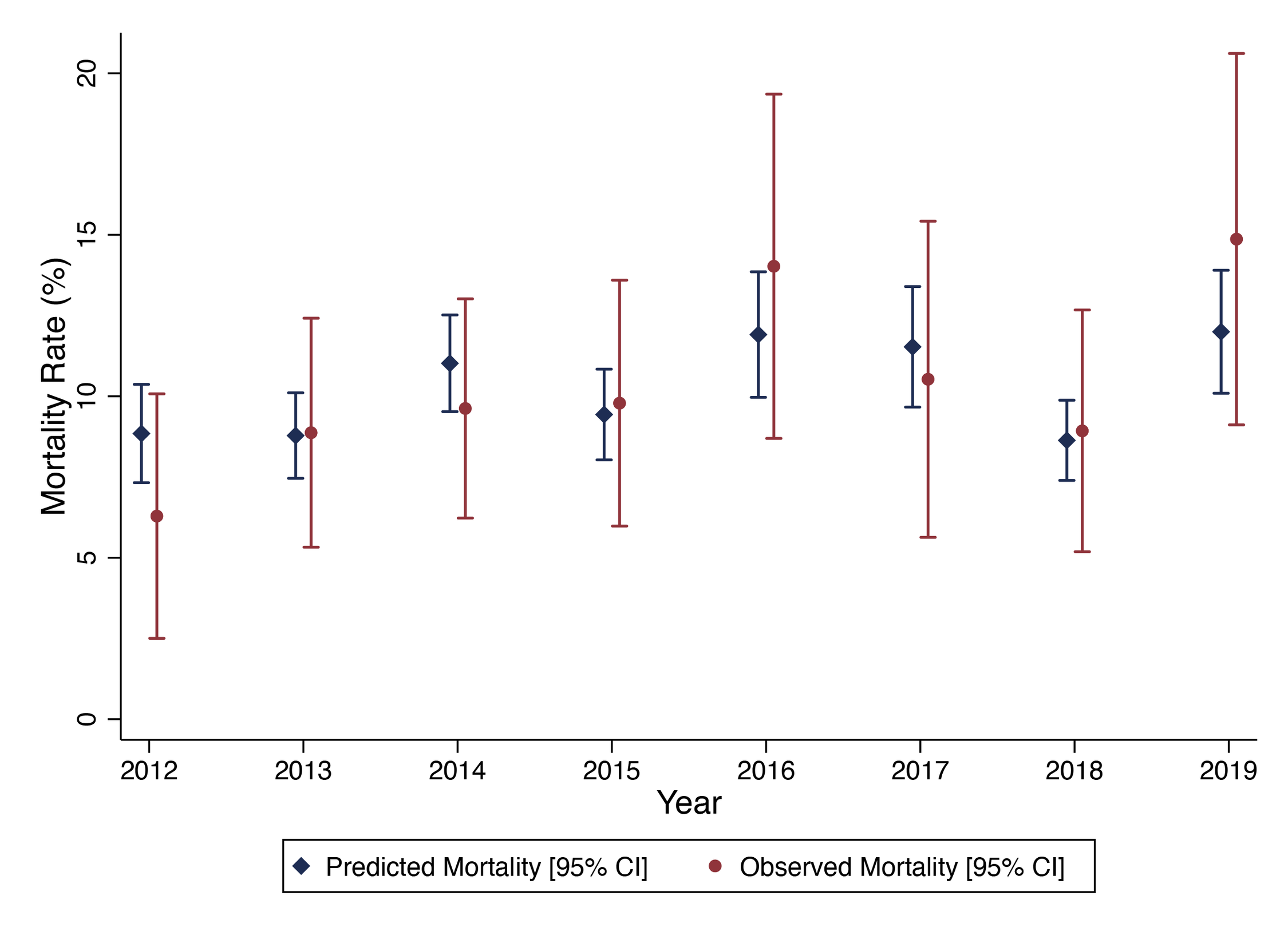

Supplement: S5 Fig — (TIF) [file pone.0264517.s005.tif]
