# Supplementary material for: Emergency care of sepsis in sub-Saharan Africa: Mortality and non-physician clinician management of sepsis in rural Uganda from 2010 to 2019
Source: PLoS One. 2022 May 11;17(5):e0264517. doi: 10.1371/journal.pone.0264517 (PMC9094533; doi:10.1371/journal.pone.0264517)
Supplement: S1 Table — (DOCX) [file pone.0264517.s006.docx]

**S1 Table. Characteristics of non-septic (qSOFA<2) and septic (qSOFA≥2) emergency unit patients with suspected infections (N = 17,490).**

|  | **No Sepsis (qSOFA < 2)** | **Sepsis (qSOFA ≥ 2)** | **p-Value** |
| --- | --- | --- | --- |
|  | **n=14,167** | **n=3,323** |  |
|  |  |  |  |
| **Age, median (IQR)** | 43 (26-68) | 40 (27-60) | <0.001^†^ |
| **Age Group** |  |  |  |
| **18-64 years old, total (%)** | 10086 (71.2) | 2642 (79.5) | <0.001 |
| **65+ years old, total (%)** | 3991 (28.1) | 661 (19.9) | <0.001 |
| **Female, total (%)** | 7327 (51.8) | 1803 (54.3) | 0.009 |
|  |  |  |  |
| **Systolic Blood Pressure, mean (95% CI)** | 119.5 (119.2 - 119.9) | 93.6 (93.1 - 94.1) | <0.001^††^ |
| **Heart Rate, mean (95% CI)** | 92.1 (91.8 - 92.5) | 105.1 (104.2 - 105.9) | <0.001^††^ |
| **Respiratory Rate, mean (95% CI)** | 21.9 (21.8 - 22.0) | 28.8 (28.6 - 29.1) | <0.001^††^ |
| **Oxygen Saturation, mean (95% CI)** | 95.3 (95.2-95.4) | 92.4 (92.1-92.7) | <0.001^††^ |
|  |  |  |  |
| **qSOFA Criteria** |  |  |  |
| **Respiratory rate ≥ 22 breaths per minute, n (%)** | 4457 (31.5) | 3207 (96.5) | <0.001 |
| **Systolic blood pressure ≤ 100 mmHg, n (%)** | 837 (5.9) | 1532 (46.1) | <0.001 |
| **Altered mentation (GCS < 15 or AVP ≠ A), n (%)** | 244 (1.7) | 441 (13.3) | <0.001 |
|  |  |  |  |
| **Co-existing Infections** |  |  |  |
| **Malaria: Smear-Positive, n (%)** | 2223 (15.7) | 690 (20.8) | <0.001 |
| **Malaria: Clinical, n (%)** | 1880 (13.3) | 467 (14.1) | 0.24 |
| **HIV, n (%)** | 1266 (8.9) | 708 (21.4) | <0.001 |
|  |  |  |  |
| **Clinician Impression, n (%)** |  |  |  |
| **"Not Sick"** | 7753 (55.1) | 951 (28.8) | <0.001 |
| **"Sick"** | 6109 (43.4) | 2178 (66.0) | <0.001 |
| **"Toxic"** | 211 (1.5) | 171 (5.2) | <0.001 |
|  |  |  |  |
| **Disposition, n (%)** |  |  |  |
| **Admitted** | 8999 (63.5) | 2927 (88.1) | <0.001 |
| **Discharged** | 5063 (35.7) | 340 (10.2) | <0.001 |
| **Expired in ED** | 47 (0.3) | 35 (1.1) | <0.001 |
| **Operating Theater** | 58 (0.4) | 21 (0.6) | 0.11 |
|  |  |  |  |
| ^†^ Wilcoxon rank-sum used as test of significance | |  |  |
| ^††^ T-test used as test of significance |  |  |  |
| All others use Fisher's exact test as test of significance | | | |
