# Supplementary material for: Emergency care of sepsis in sub-Saharan Africa: Mortality and non-physician clinician management of sepsis in rural Uganda from 2010 to 2019
Source: PLoS One. 2022 May 11;17(5):e0264517. doi: 10.1371/journal.pone.0264517 (PMC9094533; doi:10.1371/journal.pone.0264517)
Supplement: S2 Table — Dichotomous analysis was done for qSOFA<2 and qSOFA≥2. (DOCX) [file pone.0264517.s007.docx]

**S2 Table. Interventions and mortality for patients 2012-2019 stratified by qSOFA score (n=13,549).**

|  | qSOFA Score | | | | | Dichotomous qSOFA Score | | |
| --- | --- | --- | --- | --- | --- | --- | --- | --- |
|  | Zero | One | Two | Three | p-Value | No Sepsis (< 2) | Sepsis (≥ 2) | p-Value |
|  |  |  |  |  |  |  |  |  |
| **Total Cases, n** | 5855 | 5331 | 2229 | 133 | n/a | 11186 | 2362 | n/a |
| **Total Deaths, n** | 65 | 215 | 189 | 27 | n/a | 280 | 216 | n/a |
| **Crude Mortality Rate, % [95% CI]** | 1.1 [0.9 -1.4] | 4.0 [3.5 - 4.6] | 8.5 [7.4 – 9.7] | 20.3 [13.8 - 28.1] | <0.001^†^ | 2.5 [2.2 - 2.8] | 9.1 [8.0 - 10.3] | <0.001^††^ |
|  |  |  |  |  |  |  |  |  |
| **Proportion receiving interventions, (%)** |  |  |  |  |  |  |  |  |
| Neither fluids nor anti-infectives | 3565 (60.9) | 2168 (40.7) | 505 (22.7) | 18 (13.5) | 0.08^†^ | 5733 (51.3) | 523 (22.1) | <0.001** |
| Fluids only | 837 (14.3) | 1092 (20.5) | 528 (23.7) | 29 (21.8) | 0.009^†^ | 1929 (17.2) | 557 (23.6) | <0.001** |
| Anti-infectives only^*^ | 794 (13.6) | 966 (18.1) | 380 (17.1) | 16 (12.0) | 0.004^†^ | 1760 (15.7) | 396 (16.8) | 0.22** |
| Both fluids and anti-infectives | 659 (11.3) | 1105 (20.7) | 816 (36.6) | 70 (52.6) | 0.06^†^ | 1764 (15.8) | 886 (37.5) | <0.001** |
|  |  |  |  |  |  |  |  |  |
| ^†^ ANOVA used as test of significance |  |  |  |  |  |  |  |  |
| ^††^ T-test used as test of significance |  |  |  |  |  |  |  |  |
| ^*^ Anti-infectives include antibiotics, antivirals and/or antimalarials | | | |  |  |  |  |  |
| ^**^ Fisher's exact test used as test of significance | |  |  |  |  |  |  |  |
