# Supplementary material for: Emergency care of sepsis in sub-Saharan Africa: Mortality and non-physician clinician management of sepsis in rural Uganda from 2010 to 2019
Source: PLoS One. 2022 May 11;17(5):e0264517. doi: 10.1371/journal.pone.0264517 (PMC9094533; doi:10.1371/journal.pone.0264517)
Supplement: S3 Table — (DOCX) [file pone.0264517.s008.docx]

**S3 Table. Logistic regression model of mortality in septic (qSOFA≥2) patients without malaria: 2012 – 2019 (N=1,621).**

|  |  |  |  |  |  |  |  |
| --- | --- | --- | --- | --- | --- | --- | --- |
|  |  | OR | 95% CI | | | p-Value |  |
|  |  |  |  |  |  |  |  |
|  | Age |  |  |  |  |  |  |
|  | Additional Year (above 18) | 1.01 | 1.00 | - | 1.02 | 0.019 |  |
|  |  |  |  |  |  |  |  |
|  | HIV |  |  |  |  |  |  |
|  | Negative | REF |  |  |  |  |  |
|  | Positive | 1.12 | 0.7 | - | 1.7 | 0.594 |  |
|  |  |  |  |  |  |  |  |
|  | Gender |  |  |  |  |  |  |
|  | M | REF |  |  |  |  |  |
|  | F | 0.45 | 0.3 | - | 0.7 | <0.001 |  |
|  |  |  |  |  |  |  |  |
|  | Respiratory Status |  |  |  |  |  |  |
|  | Normal Rate + No Hypoxia | REF |  |  |  |  |  |
|  | Normal Rate + Hypoxia (SpO2<92%) | 1.27 | 0.4 | - | 4.0 | 0.684 |  |
|  | Tachypnea (≥22 bpm) + No Hypoxia | 1.79 | 0.4 | - | 7.5 | 0.431 |  |
|  | Tachypnea (≥22 bpm) + Hypoxia (SpO2<92%) | 4.13 | 1.3 | - | 12.9 | 0.015 |  |
|  |  |  |  |  |  |  |  |
|  | Heart Rate |  |  |  |  |  |  |
|  | Normal | REF |  |  |  |  |  |
|  | Tachycardic (≥100 bpm) | 1.56 | 1.1 | - | 2.3 | 0.022 |  |
|  |  |  |  |  |  |  |  |
|  | Temperature |  |  |  |  |  |  |
|  | Hypothermic (≤ 35.5 °C) | 2.07 | 1.4 | - | 3.1 | 0.001 |  |
|  | Normal | REF |  |  |  |  |  |
|  | Febrile (≥ 37.5 °C) | 0.59 | 0.4 | - | 0.9 | 0.024 |  |
|  |  |  |  |  |  |  |  |
|  | Blood Pressure |  |  |  |  |  |  |
|  | Not Hypotensive | REF |  |  |  |  |  |
|  | Hypotensive (SBP<100) | 1.82 | 1.3 | - | 2.6 | 0.001 |  |
|  |  |  |  |  |  |  |  |
|  | Mental Status |  |  |  |  |  |  |
|  | Normal/Not Recorded | REF |  |  |  |  |  |
|  | Altered | 1.46 | 0.9 | - | 2.3 | 0.102 |  |
|  |  |  |  |  |  |  |  |
|  | Clinical Impression |  |  |  |  |  |  |
|  | "Not Sick" | REF |  |  |  |  |  |
|  | "Sick" | 2.2 | 1.2 | - | 3.9 | 0.010 |  |
|  | "Toxic" | 6.7 | 3.2 | - | 13.8 | <0.001 |  |
|  |  |  |  |  |  |  |  |

The p-value for the Hosmer-Lemeshow goodness of fit test was 0.10, the Brier score was 0.079, and the AUROC was 0.80 (95%CI 0.77 - 0.83).
