# Supplementary material for: Emergency care of sepsis in sub-Saharan Africa: Mortality and non-physician clinician management of sepsis in rural Uganda from 2010 to 2019
Source: PLoS One. 2022 May 11;17(5):e0264517. doi: 10.1371/journal.pone.0264517 (PMC9094533; doi:10.1371/journal.pone.0264517)
Supplement: S4 Table — (DOCX) [file pone.0264517.s009.docx]

**S4 Table. Logistic Regression Model of Mortality in Septic (qSOFA≥1) Patients With Malaria: 2012 – 2019 (N=2,332).**

|  |  |  |  |  |  |  | |  | |
| --- | --- | --- | --- | --- | --- | --- | --- | --- | --- |
|  |  | Log OR | 95% CI | | | p-Value |  | |  |
|  |  |  |  |  |  |  |  | | |
|  | Age |  |  |  |  |  |  | | |
|  | Additional Year (above 18) | 1.01 | 1.00 | - | 1.02 | 0.023 |  | | |
|  |  |  |  |  |  |  |  | | |
|  | HIV |  |  |  |  |  |  | | |
|  | Negative | REF |  |  |  |  |  | | |
|  | Positive | 2.57 | 1.5 | - | 4.3 | <0.001 |  | | |
|  |  |  |  |  |  |  |  | | |
|  | Gender |  |  |  |  |  |  | | |
|  | M | REF |  |  |  |  |  | | |
|  | F | 0.76 | 0.5 | - | 1.2 | 0.220 |  | | |
|  |  |  |  |  |  |  |  | | |
|  | Respiratory Status |  |  |  |  |  |  | | |
|  | Normal Rate + No Hypoxia | REF |  |  |  |  |  | | |
|  | Normal Rate + Hypoxia (SpO2<92%) | 1.41 | 0.7 | - | 2.8 | 0.320 |  | | |
|  | Tachypnea (≥22 bpm) + No Hypoxia | 2.39 | 0.9 | - | 6.6 | 0.092 |  | | |
|  | Tachypnea (≥22 bpm) + Hypoxia (SpO2<92%) | 4.89 | 2.4 | - | 9.8 | <0.001 |  | | |
|  |  |  |  |  |  |  |  | | |
|  | Heart Rate |  |  |  |  |  |  | | |
|  | Normal | REF |  |  |  |  |  | | |
|  | Tachycardic (≥100 bpm) | 0.83 | 0.5 | - | 1.3 | 0.423 |  | | |
|  |  |  |  |  |  |  |  | | |
|  | Temperature |  |  |  |  |  |  | | |
|  | Hypothermic (≤ 35.5 °C) | 2.63 | 1.5 | - | 4.6 | 0.001 |  | | |
|  | Normal | REF |  |  |  |  |  | | |
|  | Febrile (≥ 37.5 °C) | 0.60 | 0.3 | - | 1.0 | 0.063 |  | | |
|  |  |  |  |  |  |  |  | | |
|  | Blood Pressure |  |  |  |  |  |  | | |
|  | Not Hypotensive | REF |  |  |  |  |  | | |
|  | Hypotensive (SBP<100) | 2.03 | 1.3 | - | 3.2 | 0.003 |  | | |
|  |  |  |  |  |  |  |  | | |
|  | Mental Status |  |  |  |  |  |  | | |
|  | Normal/Not Recorded | REF |  |  |  |  |  | | |
|  | Altered | 1.84 | 0.9 | - | 3.6 | 0.073 |  | | |
|  |  |  |  |  |  |  |  | | |
|  | Clinical Impression |  |  |  |  |  |  | | |
|  | "Not Sick" | REF |  |  |  |  |  | | |
|  | "Sick" | 2.9 | 1.4 | - | 5.9 | 0.004 |  | | |
|  | "Toxic" | 7.4 | 2.8 | - | 19.8 | <0.001 |  | | |
|  |  |  |  |  |  |  |  | | |

The p-value for the Hosmer-Lemeshow goodness of fit test was 0.44, the Brier score was 0.041, and the AUROC was 0.83 (95%CI 0.79 - 0.88).

Treatment of sepsis was not associated with a statistically significant increase or decrease in RR of death: “fluids alone” (RR=1.97, 95%CI 0.48 – 3.46), “anti-malarials alone” (RR=2.35, 95%CI 0.57 – 4.13),“both fluids and anti-malarials”: (RR=2.91, 95%CI 0.80 – 5.02).
