# Supplementary material for: Emergency care of sepsis in sub-Saharan Africa: Mortality and non-physician clinician management of sepsis in rural Uganda from 2010 to 2019
Source: PLoS One. 2022 May 11;17(5):e0264517. doi: 10.1371/journal.pone.0264517 (PMC9094533; doi:10.1371/journal.pone.0264517)
Supplement: S1 Text — (DOCX) [file pone.0264517.s010.docx]

The categorical variable for sepsis treatment in the emergency unit (no treatment in the emergency unit, fluids alone, antibiotics alone, or both fluids and antibiotics) was added to the logistic regression model (**S3 Table**) to assess the independent association between treatments and mortality in all septic patients without malaria. Treatment with “both fluids and antibiotics”: RR=1.85 [95%CI 1.02 – 2.69] was associated with a statistically significant *increased* relative risk (RR) of death as compared to “no treatment in the emergency unit” after controlling for the variables in the logistic regression model above. Treatment with “fluids alone” RR=1.22 [95%CI 0.57 – 1.87] and “antibiotics alone” RR=1.25 [95%CI 0.60 – 1.91] were not associated with a statistically significant difference in RR of death when compared to “no treatment in the emergency unit”.
