# Supplementary material for: Emergency care of sepsis in sub-Saharan Africa: Mortality and non-physician clinician management of sepsis in rural Uganda from 2010 to 2019
Source: PLoS One. 2022 May 11;17(5):e0264517. doi: 10.1371/journal.pone.0264517 (PMC9094533; doi:10.1371/journal.pone.0264517)
Supplement: S1 Appendix — (DOCX) [file pone.0264517.s011.docx]

**S1 Appendix. Supporting List of diagnoses defining “Suspected Infection”**

*Any diagnoses containing the following strings:*

1. Pneumonia
2. Infection
3. Fever
4. Septic/Sepsis
5. Bacteremia
6. UTI
7. Tuberculosis
8. STD
9. HIV
10. Febrile
11. Cholecystitis
12. Bronchitis
13. Colitis
14. False Tooth Extraction
15. Meningitis
16. Pelvic Inflammatory Disease
17. Peritonitis
18. Pyelonephritis
19. Malaria
20. Gastroenteritis
21. Diarrhea
22. Cellulitis
23. Dysentery
24. Tonsillitis
25. Viral Syndrome
26. Epididymitis
27. Ingrown Nail
28. Brucellosis
29. Candidiasis
30. Coryza
31. Tropical Ulcer
32. Pyomyositis
33. Giardia
34. Orchitis
35. Herpes
36. Mumps
37. Endometritis
38. Sinusitis
39. Gangrene
40. Measles
41. Hepatitis
42. Abscess
43. Appendicitis
44. Effusion
45. Pharyngitis
46. Urethritis
47. Impetigo
48. Otitis Media
49. Ludwig Angina
50. Amoebiasis
51. Mastitis
52. Prostatitis
53. Folliculitis
54. Encephalitis
55. Osteomyelitis
56. Necrotizing Fasciitis
